# Multi-omics analysis provides new insights into the changes of important nutrients and fructose metabolism in loquat bud sport mutant

**DOI:** 10.3389/fpls.2024.1374925

**Published:** 2024-03-28

**Authors:** Hai-yan Song, Ke Zhao, Yan-Gang Pei, Hong-xu Chen, Xiao-an Wang, Guo-Liang Jiang, Hong-Jiang Xie, Dong Chen, Rong-gao Gong

**Affiliations:** ^1^ College of Horticulture, Sichuan Agricultural University, Chengdu, Sichuan, China; ^2^ Horticulture Research Institute, Sichuan Academy of Agricultural Sciences, Chengdu, Sichuan, China; ^3^ Key Laboratory of Horticultural Crop Biology and Germplasm Creation in Southwestern China of the Ministry of Agriculture and Rural Affairs, Chengdu, Sichuan, China; ^4^ College of Life Science, Sichuan University, Chengdu, Sichuan, China

**Keywords:** multi-omics, bud sport, loquat (*Eriobotrya japonica* L.), fructose metabolism, nutritional value

## Abstract

Bud sport is a common and stable somatic variation in perennial fruit trees, and often leads to significant modification of fruit traits and affects the breeding value. To investigate the impact of bud sport on the main metabolites in the fruit of white-fleshed loquat, we conducted a multi-omics analysis of loquat fruits at different developmental stages of a white-fleshed bud sport mutant of Dongting loquat (TBW) and its wild type (TBY). The findings from the detection of main fruit quality indices and metabolites suggested that bud sport resulted in a reduction in the accumulation of carotenoids, fructose, titratable acid and terpenoids at the mature stage of TBW, while leading to the accumulation of flavonoids, phenolic acids, amino acids and lipids. The comparably low content of titratable acid further enhances the balanced and pleasent taste profile of TBW. Expression patterns of differentially expressed genes involved in fructose metabolism exhibited a significant increase in the expression level of *S6PDH* (*EVM0006243*, *EVM0044405*) prior to fruit maturation. The comparison of protein sequences and promoter region of *S6PDH* between TBY and TBW revealed no structural variations that would impact gene function or expression, indicating that transcription factors may be responsible for the rapid up-regulation of *S6PDH* before maturation. Furthermore, correlation analysis helped to construct a comprehensive regulatory network of fructose metabolism in loquat, including 23 transcription factors, six structural genes, and nine saccharides. Based on the regulatory network and existing studies, it could be inferred that transcription factors such as ERF, NAC, MYB, GRAS, and bZIP may promote fructose accumulation in loquat flesh by positively regulating *S6PDH*. These findings improve our understanding of the nutritional value and breeding potential of white-fleshed loquat bud sport mutant, as well as serve as a foundation for exploring the genes and transcription factors that regulate fructose metabolism in loquat.

## Introduction

Loquat (*Eriobotrya japonica* L.) is an evergreen fruit tree of the Rosaceae family, which can be classified into yellow- and white-fleshed types based on the flesh color (Jing et al., 2022). Yellow-fleshed loquat displays an orange or orange-red flesh color due to its high levels of carotenoids (Zou et al., 2020a). Moreover, yellow-fleshed varieties generally possess a denser flesh composition, thicker skin, stronger resistance to storage, and higher average fruit weight (Jiang et al., 2015a; Zou et al., 2020b). In contrast, white-fleshed loquat varieties attract consumers with their delicate creamy white or yellowish flesh, tender consistency, and succulent sweetness, which together contribute to their superior flavor profile and delectable taste that are attractive to the consumers (Zheng et al., 2017; Lin, 2019). In recent years, in order to improve the flavor and quality of loquat fruit, substantial efforts have been made to promote the development of new loquat varieties worldwide, with a particular focus on creating white-fleshed loquat germplasm (Vishal et al., 2018; Dhiman et al., 2021; Sarkar et al., 2023).

Intriguingly, our previous studies have unveiled a bud sport mutant derived from the yellow-fleshed Dongting loquat, which displayed an alluring white flesh phenotype (Sun et al., 2012, 2017). This white-fleshed mutant, known as TBW, remarkably differs from the wild type (TBY) in terms of fructose content, flesh texture, maturity, and cold tolerance, highlighting its great potential for breeding purposes (Sun et al., 2011; Li et al., 2016; Chen et al., 2017; Li et al., 2017, 2020; Pan et al., 2020). Recent studies have shown that TBW has a 321-bp deletion in the *PSY2A* gene, which encodes a rate-limiting enzyme involved in carotenoid synthesis. This genetic variation hinders the normal accumulation of carotenoids in TBW fruits (Fu et al., 2014; Song et al., 2022b). However, the specific differentially accumulated metabolites during fruit development in the white-fleshed bud sport mutant, particularly those responsible for flavor formation, remain poorly understood.

Fructose and sucrose are main carbohydrates that constitute the sweetness of loquat, and the balance between sweetness and acidity is the most important characteristic that determines the flavor of loquat (Jiang et al., 2015b). The fruits of two spontaneous mutation loquat varieties from ‘Algerí’ show suitable sweetness and high acidity, respectively (Gil et al., 2018). However, the reason for the change in the flavor of the fruits of two spontaneous mutants remains elusive. In citrus, the change of fructose content in bud sport mutants is more common (Hussain et al., 2020), and has generated many high-sweetness germplasm resources (Fang et al., 2023; Pan et al., 2023). Usually, white-fleshed loquat varieties have a higher fructose content than yellow-fleshed loquat varieties (Chen et al., 2010). However, our previous studies have shown that the fructose content in TBY fruit increases significantly before maturation, eventually leading to a significantly higher fructose content than that in TBW, suggesting that the fructose synthesis pathway of TBW is very different from that of the existing white-fleshed loquat varieties (Li et al., 2020).

Integration of metabolomic and transcriptomic analysis has been demonstrated to be valuable in deciphering the genetic and metabolic basis of somatic variations in industrial crops, including citrus, jujube, passion fruit, pepper, tomato, and sweet potato (Shi et al., 2020; Tohge et al., 2020; Zhao et al., 2022; Chen et al., 2023; Liu et al., 2023; Zheng et al., 2023a). Here, we performed a widely-targeted metabolomics analysis on the flesh during fruit development of Dongting loquat white-fleshed bud sport mutant (TBW) and its corresponding wild type (TBY). We also investigated the main internal quality indices of fruits, differentially accumulated metabolites and differentially expressed genes involved in fructose metabolic pathway. Additionally, a regulatory network of fructose metabolism in loquat centered on *S6PDH* was also revealed. Our findings provide new insights into the nutritional value of white-fleshed loquat bud sport mutant and the regulatory network of fructose metabolism in loquat.

## Materials and methods

### Plant materials and growth conditions

The white-fleshed bud sport mutant of Dongting loquat (TBW) and its corresponding wild type (TBY) were used as testing materials, which were grown in the Modern Agricultural Science and Technology Innovation Demonstration Park of Sichuan Academy of Agricultural Sciences, located at 30°46´47” N, 104°12´28” E, with an altitude of 489 m. Fruit at different developmental stages were collected at 140, 150, and 158 days after pollination (DAP), which were designated as the S1, S2, and S3 stage, respectively. To prepare samples, the flesh was obtained from ten fruits with uniform sizes. After removal of the epidermis and seeds, and the flesh was pooled into one biological replicate. Subsequently, the flesh was cut into small pieces, rapidly frozen in liquid nitrogen, and stored at –80°C. Three biological replicates were used for subsequent detection of fruit quality indices and metabolome profiling.

### Detection of main internal quality indices of fruits

The flesh were ground to a fine powder for experiments in liquid nitrogen. The content of carotenoids in the flesh was determined by the acetone-extraction method according to Song et al. (2022a). The content of soluble sugar and fructose was measured using BC0030 and BC2450 assay kits (Solarbio, Beijing, China), respectively. Titratable acid was measured using TC2303 assay kit (Leagene, Beijing, China), according to the manufacturer’s instructions.

### Metabolome profiling

For metabolome profiling, a widely-targeted metabolomics method was employed. Briefly, biological samples were freeze-dried using a vacuum freeze-dryer (Scientz-100F, Scientz, Ningbo, Zhejiang, China). The resulting freeze-dried sample was crushed using a mixer mill (MM 400, Retsch, Shanghai, China) with a zirconia bead for 1.5 min at 30 Hz. Subsequently, 50 mg of lyophilized powder was dissolved in 1.2 mL 70% methanol solution, vortexed for 30 s every 30 min for six times in total. After centrifugation at 12000 rpm for 3 min, the extracts were filtrated (SCAA-104, 0.22 μm pore size; ANPEL, Shanghai, China). The subsequent conditions of ultra-performance liquid chromatography (UPLC) and tandem mass spectrometry (MS/MS), as well as the qualitative and quantitative analysis of metabolites, have been described in the experimental steps of Ding et al. (2023).

### Screening and enrichment analysis of differentially accumulated metabolites

Principal component analysis (PCA) was performed on different metabolome samples using the prcomp in R (Shu et al., 2023). Differentially accumulated metabolites (DAMs) were identified by filtering with∣Log_2_ fold change∣≥ 1 and *p*-value < 0.05. The identified metabolites were annotated using the Kyoto Encyclopedia Genes and Genomes (KEGG) compound database (http://www.kegg.jp/kegg/compound/), and the annotated metabolites were then mapped to the KEGG pathway database (http://www.kegg.jp/kegg/pathway.html).

### Analysis of expression patterns of key genes involved in fructose metabolic pathway

The RNA-seq data for different developmental stages of TBY and TBW were obtained from the NGDC repository (https://ngdc.cncb.ac.cn/gsa) with the accession number of CRA011296. A local database was constructed using the published loquat genome of Seventh star (Jiang et al., 2020a). Key genes involved in the fructose metabolism pathway in loquat were identified using BlastP following the method of Su et al. (2021). Pathway maps illustrating the key metabolites and genes related to fructose metabolism were generated using TBtools (Chen et al., 2020) and Adobe Illustrator 2021. Total RNA extraction and quantitative real-time polymerase chain reaction (qRT-PCR) analysis were conducted according to the protocol outlined by Song et al. (2022a). Total RNA extraction from samples was performed using the trizol extraction method. The qRT-PCR procedure followed the SYBR^®^ Premix Ex Taq manual (Takara, Dalian, Liaoning, China). The relative transcript level of each gene was calculated using the 2^-ΔΔCt^ method. Primers used in qRT-PCRs are listed in **Supplementary Table S1**.

### Resequencing, protein sequence alignment and *cis*-acting element prediction of 2000 bp upstream of the coding region

Sufficient young leaves of TBY and TBW were collected for nanopore resequencing with the average depth of 30× to 40× following the standard protocol provided by Oxford Nanopore Technologies, including sample quality testing, library construction, library quality testing, and library sequencing (Deamer et al., 2016; Jain et al., 2016). The obtained clean reads were then mapped to the reference genome (Jiang et al., 2020a) using Minimap2 (Li, 2018). The gene sets of TBY and TBW were annotated in Non-Redundant Protein Sequence Database (Deng et al., 2006), SwissProt (Apweiler et al., 2004), Gene Ontology (Ashburner et al., 2000), KEGG (Kanehisa et al., 2004), and Pfam (Finn et al., 2014) databases by BLAST. Homologous genes of *EVM0006243* (*S6PDH*) and *EVM0044405* (*S6PDH*) in TBY, TBW, Jiefangzhong (Su et al., 2021) and Seventh star (Jiang et al., 2020a) were obtained through BlastP. Subsequently, DNAMAN version 9.0 (https://www.lynnon.com) was used to compare the protein sequences encoded by the genes in different loquat materials. The 2000 bp sequence upstream of the coding region was extracted using TBtools (Chen et al., 2020), and the *cis*-acting elements were predicted using PlantCARE (http://bioinformatics.psb.ugent.be/webtools/plantcare/html/).

### Transcription factor prediction based on loquat transcripts

The Plant Transcription Factor Database (PlantTFDB, http://planttfdb.gao-lab.org/) and iTAK (Zheng et al., 2016) were used to annotate all transcription factors (TFs) identified in the RNA-seq data obtained from the flesh tissues of loquat at different developmental stages. mRNA expression levels were determined by calculating the number of fragments per kilobase of transcript per million fragments mapped (FPKM) and the log_2_(FPKM) values of all TFs were then used to generate heat map using TBtools (Chen et al., 2020).

### Construction of the potential regulatory network of fructose in loquat

Based on the transcriptomic and metabolomic data, a correlation analysis was performed using the Metware Cloud, an online platform for data analysis (https://cloud.metware.cn), by employing the Pearson correlation calculation method. The top 20 TFs with the highest correlation values with *EVM0006243* or *EVM0044405* were screened out respectively and combined. Pearson correlation coefficients were calculated to measure the degree of association between genes and metabolites for 16 differentially accumulated saccharides, 10 differentially expressed genes (DEGs) with high expression levels involved in fructose metabolism, and 23 TFs obtained in the previous step. DAMs and DEGs that could not be linked to the main metabolic network were filtered out, and the resulting correlation network plots were generated using Cytoscape v3.9.1 (Otasek et al., 2019).

## Results

### Detection of main fruit internal quality indices and metabolite profiling of TBY and TBW

**Figure 1:**
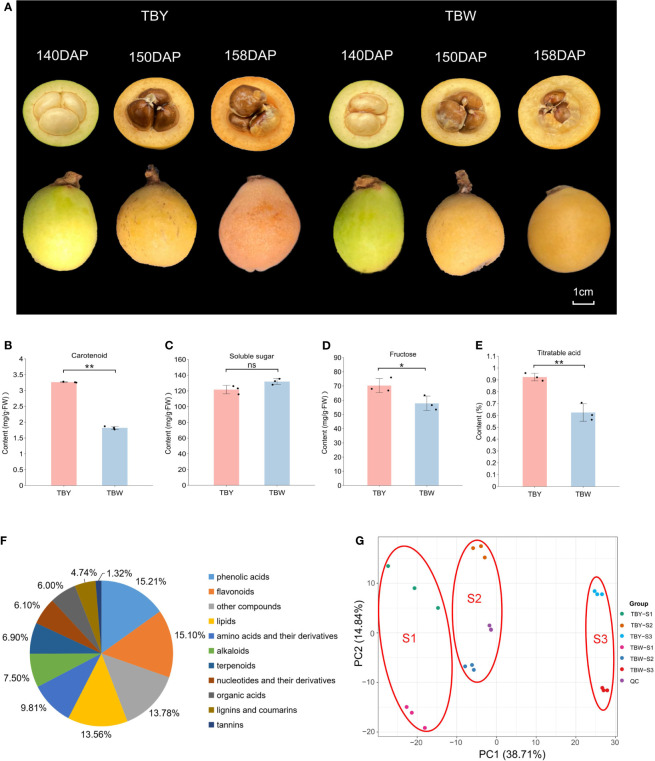
Detection of main fruit internal quality indices and metabolite profiling of TBY and TBW. **(A)** Phenotype of TBY and TBW at different fruit developmental stages. Intact and sectioned loquats were photographed at 140, 150, and 158 days after pollination (DAP). **(B-E)** Contents of carotenoids, soluble sugars, fructose and titratable acid in ripe fruits of TBY and TBW. One or two asterisks indicate statistical significance by Student’s *t*-tests at 0.05 or 0.01 levels, respectively. NS represents not significant. **(F)** Pie graph of total metabolites in all samples. **(G)** Principal component analysis for metabolomes.

Previous studies have shown that TBY and TBW show great differences in fruit quality and flavor at mature stage (Li et al., 2020). Therefore, fruits of three key developmental stages of TBY and TBW were collected and the main quality indices in flesh at mature stage were detected (**Figure 1A**). Compared with TBY, the contents of carotenoids, fructose and titratable acid in the flesh of TBW were significantly reduced, but there was no difference in soluble sugar, which made a higher sugar-acid ratio and better flavor of TBW (**Figures 1B–E**).

UPLC-MS/MS technology was employed to analyze the metabolites in TBY and TBW at different developmental stages. A total of 907 metabolites were identified, including 138 phenolic acids, 137 flavonoids, 123 lipids, 89 amino acids and their derivatives, 68 alkaloids, 63 terpenoids, 55 nucleotides and their derivatives, 54 organic acids, 43 lignins and coumarins, 12 tannins, and 125 other unclassified metabolites (**Figure 1F**, **Supplementary Table S2**). Notably, out of the 125 other metabolites, 60 were classified as saccharides after secondary classification, ranking saccharides as the fourth most abundant metabolites among all the identified metabolites.

PCA was performed on the samples of TBY and TBW at different developmental stages (**Figure 1G**). The metabolome samples were distinctly separated into six groups. The samples from different stages were clearly clustered into three groups on PC1, which accounted for 38.71% of the total variation. Additionally, the two test materials were distinctly separated into two groups on PC2, which explained 14.84% of the total variation. These results suggested that both genotype and fruit developmental stage have great impacts on the accumulation of metabolites in the flesh of loquat.

### Screening and analysis of differentially accumulated metabolites

**Figure 2:**
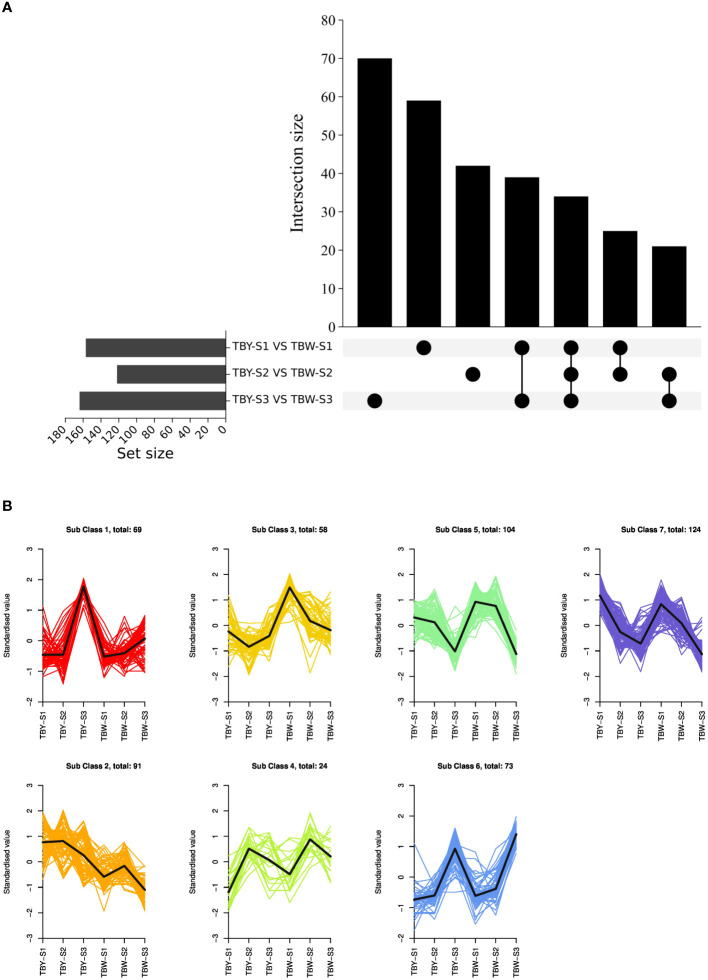
Analysis of differentially accumulated metabolites (DAMs). **(A)** Upset plots of ubiquitously and exclusively DAMs in pairwise comparisons across developmental stages between TBY and TBW. The set size at the lower left shows the number of metabolites contained in each dataset. The dots at the lower right refer to the corresponding data set on the left side through the horizontal correspondence. The vertical point-to-point connection represents the overlap of the corresponding data sets, and the number of metabolites in the intersection is displayed by the intersection size at the top. **(B)** K-means clustering of all the detected DAMs. The expression profiles of metabolites in each cluster are represented in different colors, and the average expression profiles of all metabolites in each sample are represented in black.

To investigate the potential influence of bud sport on the accumulation of DAMs during fruit development of loquat, we analyzed the metabolome data of TBY and TBW at the same developmental stage (**Figure 2A**). The number of DAMs between TBY and TBW was 158, 123, and 165 at the S1, S2, and S3 stage, respectively. At the S1 stage, there were 74 up-regulated and 84 down-regulated metabolites in TBW relative to those in TBY (**Supplementary Table S3**), whereas at the S2 stage, there were 84 up-regulated and 39 down-regulated metabolites (**Supplementary Table S4**). In contrast, at the S3 stage, there were more down-regulated metabolites (128) than up-regulated metabolites (37) (**Supplementary Table S5**). Notably, in mature fruits, TBW had significantly higher contents of 10 phenolic acids, nine amino acids and seven flavonoids, while significantly lower contents of 39 triterpenes than TBY.

For different developmental stages of the same material, there were 73 up-regulated and 93 down-regulated DAMs from the S1 to S2 stage in TBY (**Supplementary Table S6**), whereas the numbers in TBW were 81 and 48 (**Supplementary Table S7**), respectively. Subsequently, there were 134 up-regulated and 140 down-regulated DAMs from the S2 to S3 stage in TBY (**Supplementary Table S8**), whereas the numbers were 256 and 81 in TBW, respectively (**Supplementary Table S9**). 16 differentially accumulated saccharides were identified at all three stages between TBY and TBW (**Supplementary Figure S1**, **Supplementary Table S10**). Among them, the numbers of up-regulated and down-regulated saccharides from the S1 to S2 stage were both three in TBY, while there were eight up-regulated saccharides from the S2 to S3 stage. In TBW, there were one up-regulated and seven down-regulated saccharides from the S1 to S2 stage, and two down-regulated saccharides from the S2 to S3 stage. Overall, TBY and TBW showed accelerated accumulation of various saccharides along with fruit development.

To further investigate the accumulation pattern of major DAMs during fruit development in TBY and TBW, the DAMs were divided into seven modules by K-means clustering (**Figure 2B**, **Supplementary Table S11**). We found that 16 differentially accumulated saccharides were clustered into modules 1, 5, 6, and 7, which comprised 4, 1, 8, and 3 saccharides, respectively. Among them, module 1 exhibits a high similarity to the previously reported fructose accumulation pattern of TBY and TBW (Li et al., 2016, 2017), and there are four saccharides within this module, including sorbitol-6-phosphate, sedoheptulose, DMelezitose o-rhamnoside and D-Melezitose These results suggested that the difference in fructose content during fruit development between TBY and TBW could be attributed to the differential accumulation of these 4 saccharides.

### KEGG enrichment and clustering analysis of DAMs

KEGG enrichment analysis was performed on the DAMs identified at the S1 stage between TBY and TBW (**Supplementary Figure S2**). The DAMs were successfully annotated into 40 pathways in the KEGG database, and the top five enriched pathways included metabolic pathways (23 DAMs), biosynthesis of secondary metabolites (12 DAMs), biosynthesis of cofactors (6 DAMs), 2-oxycarboxylic acid metabolism (5 DAMs), pyrimidine metabolism (4 DAMs), and biosynthesis of amino acids (4 DAMs). Additionally, the DAMs between TBY and TBW at S2 stage were annotated to 43 pathways (**Supplementary Figure S3**), and the top three enriched pathways were consistent with those observed at the S1 stage. Furthermore, DAMs related to the biosynthesis of flavone and flavonol, as well as purine metabolism, were found to increase in the flesh of TBW from S2 stage.

**Figure 3:**
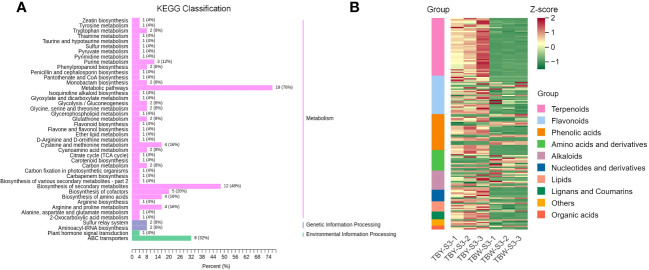
KEGG enrichment **(A)** and cluster analysis **(B)** of DAMs between TBY and TBW at S3 stage. The content of metabolites in each row of the clustering heatmap is standardized using Z-score values. All DAMs detected at the S3 stage are sorted according to classification and number, then labeled with different colors on the left side of the heatmap.

Notably, there were significant differences in enriched pathways of DAMs between TBY and TBW at the S3 stage (**Figure 3A**). A large number of DAMs involved in amino acid metabolic pathways were enriched in TBW, specifically in pathways such as cysteine and methionine metabolism (4 DAMs), biosynthesis of amino acids (4 DAMs), and arginine and proline metabolism (4 DAMs). To further explore the overall profile of DAMs at the S3 stage, a clustering analysis was performed (**Figure 3B**, **Supplementary Table S12**) The cluster heatmap showed that there were 10 distinct categories of DAMs at the S3 stage between TBY and TBW. Among them, the content of terpenoids in the mature flesh of TBW was significantly lower than that of TBY, and the significantly up-regulated metabolites were concentrated in flavonoids, phenolic acids, amino acids and lipids. These results can serve as an important support for future studies on the differences in flavor and nutrient composition between TBY and TBW.

### Expression pattern of key genes involved in fructose metabolism of loquat

**Figure 4:**
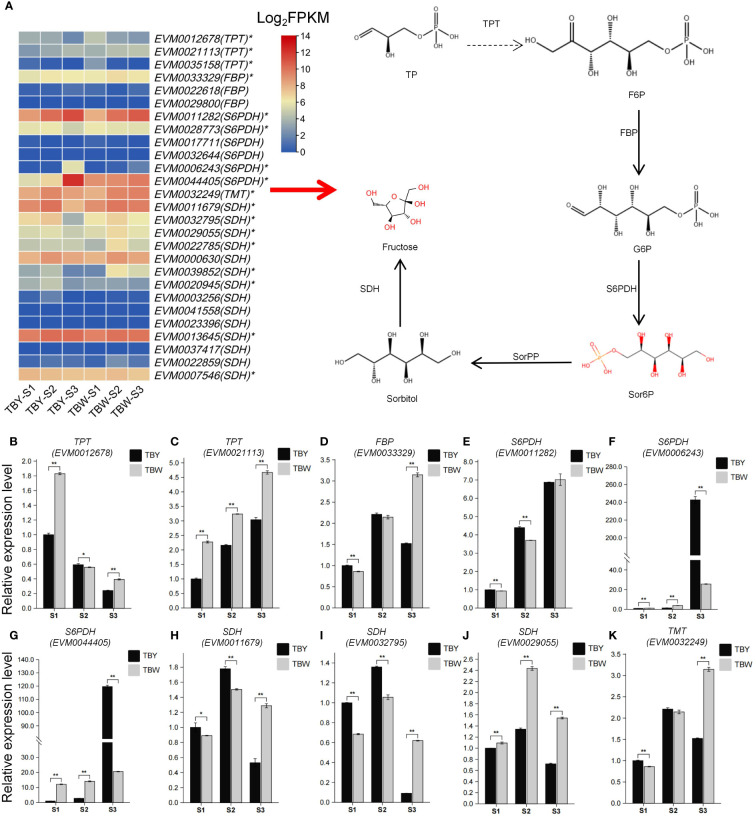
Key genes involved in fructose metabolism of loquat and their expression patterns. **(A)** Heatmap of 27 genes involved in fructose metabolism pathways. * represents differentially expressed genes at the *p*°CxFF1C;0.05 level by Student’s *t*-test. TP, Triose phosphate; TPT, Triose phosphate transporter; F6P, Fructose-6-phosphate; FBP, Fructose bisphosphatase; G6P, Glucose-6-phosphate; S6PDH, Sorbitol 6-phosphate dehydrogenase; Sor6P, Sorbitol 6-phosphate; SorPP, Sorbitol 6-phosphate phosphatase; SDH, Sorbitol dehydrogenase; **(B-K)** qRT-PCR verification of 10 highly expressed fructose metabolism-related differentially expressed genes. One or two asterisks indicate statistical significance by Student’s *t*-tests at 0.05 or 0.01 levels, respectively.

A total of 27 genes associated with the fructose metabolism pathway were identified from the reference genome by using BlastP, including three *triose phosphate transporter* (*TPT*), three *fructose bisphosphatase* (*FBP*), six *sorbitol 6-phosphate dehydrogenase* (*S6PDH*), 14 *sorbitol dehydrogenase* (*SDH*), and one *tonoplast monosaccharide transporter* (*TMT*), among which 17 genes were differentially expressed genes (**Figure 4A**, **Supplementary Table S13**). Subsequently, the expression patterns of 10 highly expressed DEGs were analyzed by qRT-PCR (**Figures 4B–K**). The results showed that six genes, namely *TPT* (*EVM0021113*), *FBP* (*EVM0033329*), *S6PDH* (*EVM0011282*, *EVM0006243*, *EVM0044405*), and *TMT* (*EVM0032249*), exhibited gradual increases in expression with the development of loquat fruit. Conversely, *TPT* (*EVM0012678*) showed a gradually decreasing trend in expression, and *SDH* (*EVM0011679*, *EVM0032795*, *EVM0029055*) first exhibited an increasing trend followed by a decreasing trend. Notably, TBY had significantly higher expression levels of *S6PDH* (*EVM0006243*, *EVM0044405*), which were 9.5-fold and 5.8-fold those of TBW (**Figure 4G, H**). The expression of *S6PDH* (*EVM0006243*, *EVM0044405*) in TBY showed a sharp increase before fruit maturation, which may lead to a rapid increase in fructose content in flesh.

### Key transcription factors involved in fructose accumulation in loquat and potential regulatory network

**Figure 5:**
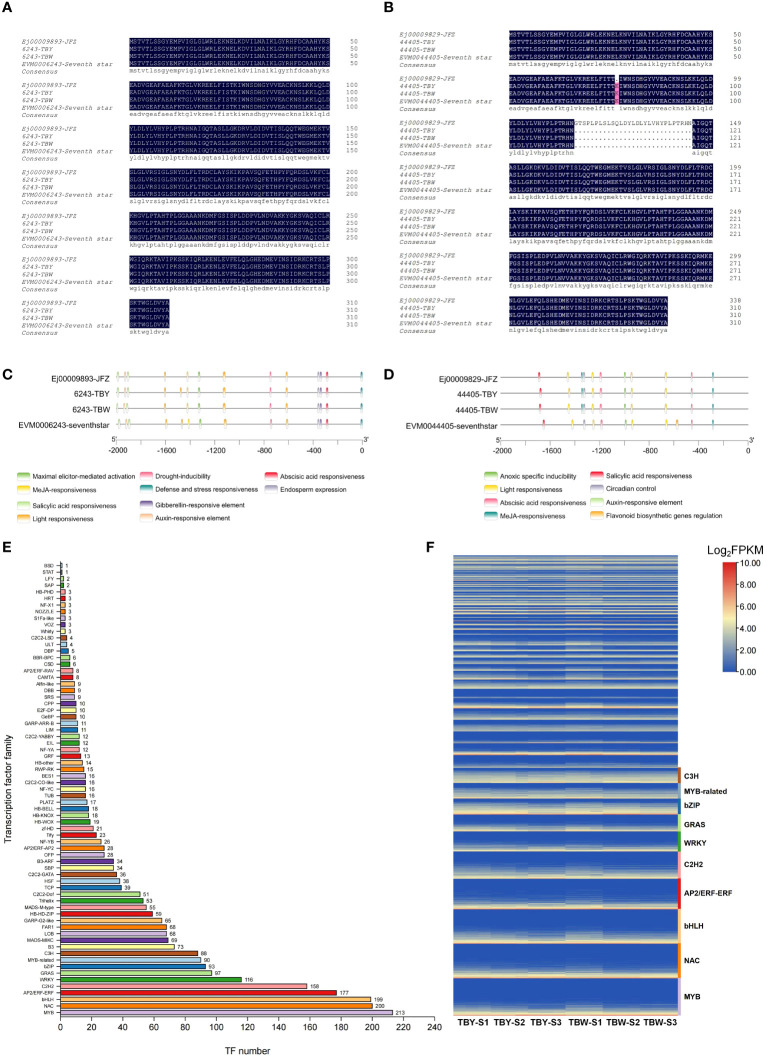
The comparison of protein sequences and promoter region of *S6PDH* (*EVM0006243*, *EVM0044405*) between TBY and TBW indicated that transcription factors may be responsible for the rapid up-regulation of *S6PDH* before maturation **(A)** The protein sequences encoded by *EVM0006243* in four loquat materials were aligned. JFZ represents the sequence from yellow-fleshed loquat ‘Jiefangzhong’, and ‘Seventh star’ represents the sequence from a white-fleshed loquat variety, the same below. **(B)** The protein sequences encoded by *EVM0044405* in four loquat materials were aligned. **(C)** The *cis*-acting elements in four loquat materials of the 2000 bp region upstream of the coding region of *EVM0006243*. **(D)** The *cis*-acting elements in four loquat materials of the 2000 bp region upstream of the coding region of *EVM0044405.* **(E)** Bar chart of transcription factor families annotated from RNA-seq data of loaquat. The number of each transcription factor family is displayed in a bar. **(F)** Heat map of expression levels of different transcription factor families in flesh of loquat. Genes in each transcription factor family are ranked according to the average expression levels from low to high, and the top 10 transcription factor families are labeled by different colors on the right side.

The protein sequences encoded by *S6PDH* (*EVM0006243*, *EVM0044405*) of four distinct loquat materials were aligned (**Figures 5A, B**). The results demonstrated that the protein sequences encoded by *EVM0006243* were completely consistent across all four materials, whereas the protein sequences of *EVM0044405* showed slight structural variation in the ‘Jiefangzhong’ material. Further analysis of the promoter region (2000 bp upstream of the initiator codons) of *S6PDH* (*EVM0006243*, *EVM0044405*) revealed the distribution of various common *cis*-acting elements upstream of the coding region (**Figures 5C, D**). Interestingly, the promoter region of *EVM0006243* contains four light-responsive *cis*-acting elements in TBY, but only three in TBW. Taken together, there are no structural variations in protein sequences or promoter region of *S6PDH* between TBY and TBW that affects gene function or expression. These results indicated that the reason for the different expression patterns of *S6PDH* (*EVM0006243*, *EVM0044405*) in TBY and TBW may be upstream transcription factors, rather than gene structural variation.

**Figure 6:**
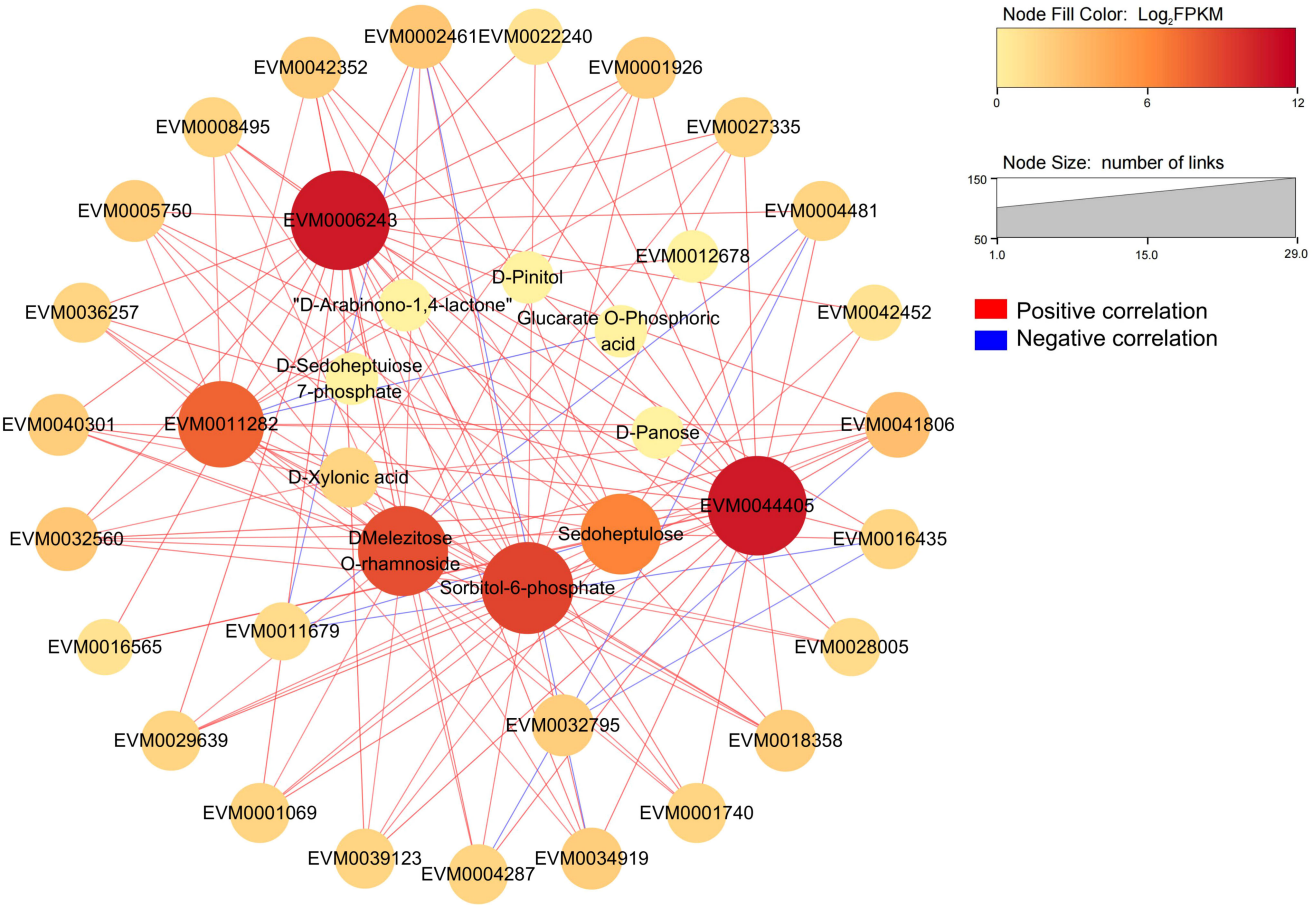
The correlation network of key structural genes, transcription factors and metabolites in the fructose metabolic pathway of loquat. 7 highly expressed structural genes related to fructose metabolism are located in the middle of the circle. 23 transcription factors and 7 saccharides related to fructose metabolism are located in the outer and inner circle, respectively.

RNA-seq data annotated a total of 2660 transcription factors (TFs) belonging to 69 families, and the top 10 TF families were myeloblastosis (213 MYB TFs), NAM/ATAF1/2/CUC2 (200 NAC TFs), basic helix–loop–helix (199 bHLH TFs), APETALA2 ethylene response factor/ethylene response factor (177 AP2ERF/ERF TFs), Cys2-His2 (158 C2H2 TFs), WRKY (116 TFs), GAI-RGA- and -SCR (97 GRAS TFs), basic leucine zipper (91 bZIP TFs), MYB-related (90 TFs), and Cysteine3Histidine (88 C3H TFs) (**Figure 5E**, **Supplementary Table S14**). The global expression levels of these TFs during loquat fruit development are shown in **Figure 5F**, with members of C3H, bZIP, and MYB-related families being highly expressed during fruit development. Interestingly, the bZIP family had the most members that were correlated with *S6PDH* (*EVM0006243*, *EVM0044405*) with correlations coefficients above 0.6 (**Supplementary Table S15**, **Supplementary Table S16**), indicating that the bZIP family may play a critical role in loquat fruit development and fructose accumulation. By combining the top 20 TFs with the highest correlation with *S6PDH*, a total of 23 TFs were obtained (**Table 1**). Subsequently, a potential regulatory network was constructed by combining these 23 TFs with highly expressed structural genes and differentially accumulated saccharides (**Figure 6**).

**Table 1:** The combined transcription factors with the highest correlation with *S6PDH (EVM0006243* and *EVM0044405)*.

No.	Transcription factor	Gene Family	Structural gene (*EVM0006243*)		Structural gene (*EVM0044405*)	
			Correlation	P-value	Correlation	P-value
1	*EVM0039123*	GARP-G2-like	0.9903	3.94759E-15	0.9881	1.98895E-14
2	*EVM0001069*	AP2/ERF-ERF	0.9878	2.30468E-14	0.9730	1.29771E-11
3	*EVM0029639*	AP2/ERF-ERF	0.9876	2.76743E-14	0.9791	1.72407E-12
4	*EVM0016565*	NAC	0.9786	2.0602E-12	0.9685	4.43664E-11
5	*EVM0032560*	C3H	0.9736	1.09304E-11	0.9794	1.51974E-12
6	*EVM0040301*	LOB	0.9694	3.5101E-11	0.9868	4.37761E-14
7	*EVM0036257*	MYB-related	0.9687	4.19426E-11	0.9724	1.53231E-11
8	*EVM0005750*	GRAS	0.9641	1.25193E-10	0.9853	1.07286E-13
9	*EVM0008495*	bZIP	0.9629	1.59971E-10	0.9653	9.56075E-11
10	*EVM0042352*	C2H2	0.9628	1.64052E-10	0.9701	2.90002E-11
11	*EVM0002461*	B3-ARF	0.9609	2.42848E-10	0.9738	1.04255E-11
12	*EVM0022240*	C3H	0.9591	3.43458E-10	0.9743	8.7138E-12
13	*EVM0001926*	C2H2	0.9575	4.64147E-10	0.9806	9.38534E-13
14	*EVM0027335*	EIL	0.9573	4.84562E-10	0.9819	5.47831E-13
15	*EVM0004481*	HB-BELL	0.9565	5.63459E-10	0.9567	5.37761E-10
16	*EVM0042452*	GRAS	0.9553	6.90288E-10	0.9228	4.98433E-08
17	*EVM0041806*	NAC	0.9547	7.73119E-10	0.9680	4.94839E-11
18	*EVM0016435*	HB-HD-ZIP	0.9456	3.24188E-09	0.9400	7.00512E-09
19	*EVM0028005*	B3	0.9417	5.56934E-09	0.9574	4.77966E-10
20	*EVM0018358*	MYB-related	0.9401	6.90114E-09	0.9391	7.83826E-09
21	*EVM0001740*	B3-ARF	0.9285	2.72691E-08	0.9692	3.71487E-11
22	*EVM0034919*	PLATZ	0.9348	1.34358E-08	0.9657	8.60288E-11
23	*EVM0004287*	BBR-BPC	0.9198	6.64545E-08	0.9576	4.5805E-10

## Discussion

Bud sport selection is a unique breeding approach for perennial and asexually propagated fruit trees, particularly those with long juvenile periods, such as loquat and citrus (Wang et al., 2021a). This approach may lead to dramatic changes in horticultural traits of fruit trees, such as fruit size, flavor, maturity, and resistance (He et al., 2022; Niu et al., 2022; Zhou et al., 2023; Zhang et al., 2023a). Through analysis of the mechanism for bud sport mutation and utilization of bud sport mutants, abundant fruit types have been created, such as high anthocyanin blood orange, red and easy coloring apple, purple-peeled fig, non-climacteric Japanese plum and peach (Butelli et al., 2012; Gu et al., 2016; Farcuh et al., 2017; Wang et al., 2017; Jiang et al., 2020b). Therefore, it is of great significance to analyze the mechanism for quality formation in fruit tree mutant materials with significant phenotypic changes and high breeding value.

Typically, fruits of white-fleshed loquat are smaller, lack of carotenoids, and exhibit weaker stress resistance compared to yellow-fleshed loquat, which limits our understanding of their nutritional value and breeding potential (Fu et al., 2014; Li et al., 2017; Peng et al., 2022). In the present study, bud sport resulted in significantly lower contents of carotenoid, fructose and titratable acid in the mature flesh of TBW compared to TBY. But the relatively low titratable acid content contributes to a more balanced and pleasant taste profile of TBW, making it a desirable variety for consumers. More interestingly, TBW demonstrated the capacity to accumulate a certain amount of carotenoids in its mature flesh, which is far higher than that of the existing white-fleshed loquat varieties (Liu et al., 2016). The white flesh trait in loquat has been confirmed to be controlled by a single recessive gene, which leads to the inability of normal accumulation of carotenoids in white-fleshed loquats (Fu et al., 2014; Zou et al., 2020a; Song et al., 2022b). However, our findings suggest that bud sport did not completely block the synthesis of carotenoids in the flesh of TBW, which enhanced its nutritional value and breeding potential.

To further elucidate the impact of bud sport on the major metabolites in loquat fruits, we constructed the metabolome profiles of TBY and TBW at different developmental stages by widely-targeted metabolomics. Bud sport led to a significant decrease in terpenoids in the mature flesh of TBW, while flavonoids, phenolic acids, amino acids and lipids accumulated abundantly. In the bud sport apples, the genes involved in the terpenoid biosynthesis pathway were significantly down-regulated, while those related to the flavonoid biosynthesis pathway were up-regulated, ultimately leading to the accumulation of anthocyanins (Li et al., 2018). However, the relationship between the terpenoid and flavonoid metabolic pathways remains to be further verified through additional gene function experiments. In fact, carotenoids also belong to terpenoids, and there have been extensive studies on the relationship between the carotenoid and flavonoid biosynthesis pathways. A recent study on the pigments of wild loquats and cultivated varieties has demonstrated that the content of carotenoids and flavonoids in the flesh displays a significant opposing trend in the process of evolution, indicating a potential metabolic flux in the fruit (Su et al., 2023). Analogously, the flavonoid and carotenoid biosynthetic pathways affect fruit quality by competing for metabolic flux in *SlPSY1* loss-of-function mutants of tomato (Cao et al., 2023), which is the same as the effect of *PSY2A* deletion in TBW in our previous study (Song et al., 2022b). Thus, our findings reinforce the notion that flavonoids play a crucial role in bud sport and plant evolution, which may compete with carotenoids for metabolic flux in fruits.

The sugar accumulation in loquat can be categorized into fructose accumulation type, hexose (fructose and glucose) accumulation type, and sucrose accumulation type (Jiang et al., 2015b). The content of fructose varies significantly among different varieties of loquat and is recognized as a crucial component in determining the flavor profile of loquat (Chen et al., 2010; Li et al., 2017). Loquat exhibited a distinctive pattern of rapid fructose accumulation and acid reduction before fruit maturation, but the key genes regulating this process have rarely been reported (Chen et al., 2010; Jiang et al., 2015b; Li et al., 2020). Previous studies have revealed that single nucleotide polymorphisms (SNPs) in *NAD^+^-SDH* (a gene encoding a rate-limiting enzyme in the sorbitol metabolic pathway) of loquat may influence its expression, thereby affecting the accumulation of fructose (Li et al., 2016). However, the expression level of *NAD^+^-SDH* increased slowly before fruit maturation, which could not explain the rapid accumulation of fructose in loquat. Here, we identified 16 differentially accumulated saccharides during fruit development in the metabolomics data, among which sorbitol-6-phosphate is a key compound in the biosynthetic pathway of fructose. The significant up-regulation of sorbitol-6-phosphate during the S2 to S3 stages in TBY accounted for the significantly higher fructose content in the mature fruits of TBY compared to TBW, and *S6PDH* (*EVM0006243*, *EVM0044405*) may be the key gene involved in this process. *S6PDH* has been identified as a key gene involved in the sorbitol and fructose biosynthetic pathway in fruits of the Rosaceae family (Wu et al., 2013; Shen et al., 2017), but there have been no reports on its gene function in loquat so far. Our investigation provides a research foundation for exploring the key genes involved in fructose metabolism pathway of loquat.

**Figure 7:**
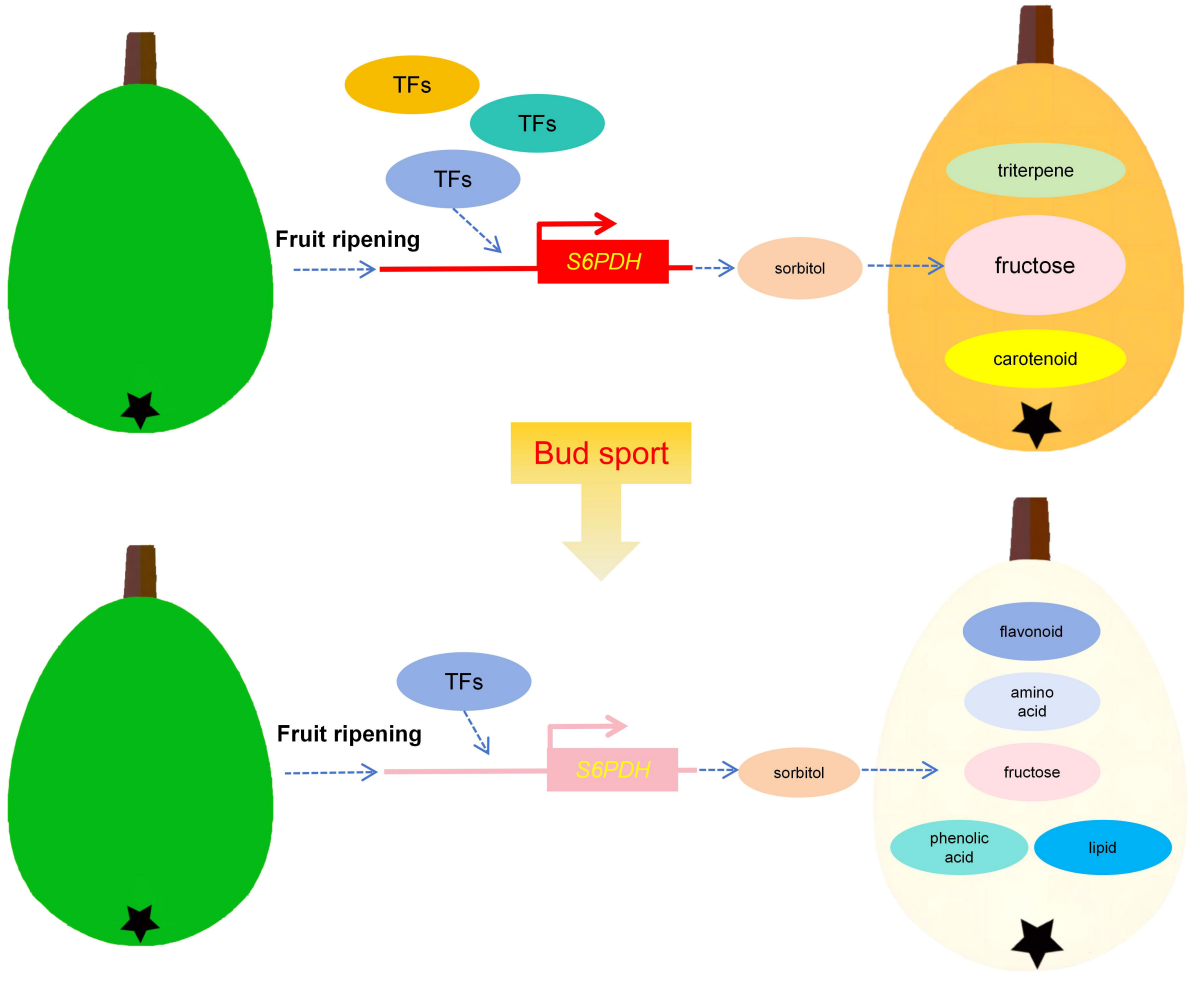
Model of differences in the accumulation of major metabolites during fruit development between white-fleshed bud sport Dongting loquat (TBW) and its wild-type(TBY).

The accumulation of carbohydrates in fruits is synergistically regulated by various TFs (Ren et al., 2023). However, due to the delayed publication of the loquat genome and the absence of a stable gene function verification system, there have been few reports on TFs associated with the sugar metabolism pathway in loquat (Xu et al., 2023). In this study, the comparison of protein sequences and promoter region of *S6PDH* (*EVM0006243*, *EVM0044405*) between TBY and TBW showed that there was no structural variation affecting gene function or expression, indicating that TFs may be responsible for the rapid up-regulation of *S6PDH* before maturation. Through correlation analysis of expression levels, we identified 23 TFs highly related to fructose metabolism, including ERF, NAC, MYB, GRAS, and bZIP family members, which have been extensively studied for their involvement in regulating fruit sucrose accumulation (Wang et al., 2021b; Zhang et al., 2022; Shu et al., 2023; Zhang et al., 2023b; Zheng et al., 2023b). These novel insights can serve as a significant stepping stone for future studies aiming to unravel the key TFs upstream of *S6PDH* in loquat. Based on the above results, we proposed a model to illustrate the impact of bud sport on important nutrients and fructose accumulation in loquat (**Figure 7**).

## Conclusion

In this study, we employed multi-omics analysis to gain a comprehensive understanding of the metabolic changes in loquat bud sport mutant. Our findings reveal that bud sport reduces the accumulation of fructose, carotenoids titratable acid and terpenoids at the mature stage of TBW, whereas leads to the flow of metabolites to flavonoids, phenolic acids, amino acids and lipids. Through excavation of transcriptomic data and analysis of expression patterns, *S6PDH* (*EVM0006243*, *EVM0044405*) was identified as a key candidate gene leading to the rapid accumulation of fructose in loquat before maturation, which may be regulated by a variety of transcription factor families such as ERF, NAC, MYB, GRAS, and bZIP. These results not only improve our understanding of the nutritional value and breeding potential of loquat bud sport mutants, but also provide candidate genes and potential transcription factors that regulate fructose accumulation in loquat.

## Data availability statement

The nanopore sequencing data presented in the study are deposited in the NGDC repository (https://ngdc.cncb.ac.cn/gsa), accession number CRA014295.

## Author contributions

H-YS: Conceptualization, Data curation, Formal analysis, Funding acquisition, Investigation, Methodology, Project administration, Resources, Software, Supervision, Validation, Visualization, Writing – original draft, Writing – review & editing. KZ: Conceptualization, Data curation, Formal analysis, Investigation, Methodology, Project administration, Software, Supervision, Validation, Visualization, Writing – original draft, Writing – review & editing. Y-GP: Conceptualization, Formal analysis, Investigation, Software, Writing – review & editing. H-XC: Formal analysis, Investigation, Software, Writing – review & editing. X-AW: Conceptualization, Formal analysis, Software, Writing – review & editing. G-LJ: Investigation, Resources, Supervision, Writing – review & editing. H-JX: Investigation, Resources, Supervision, Writing – review & editing. DC: Conceptualization, Funding acquisition, Investigation, Resources, Supervision, Writing – original draft, Writing – review & editing. RG-G: Conceptualization, Formal analysis, Investigation, Supervision, Writing – original draft, Writing – review & editing.
