# Supplementary material for: Multi-omics analysis provides new insights into the changes of important nutrients and fructose metabolism in loquat bud sport mutant
Source: Front Plant Sci. 2024 Mar 28;15:1374925. doi: 10.3389/fpls.2024.1374925 (PMC11008694; doi:10.3389/fpls.2024.1374925)
Supplement: Supplementary file 2 [file DataSheet_2.docx]

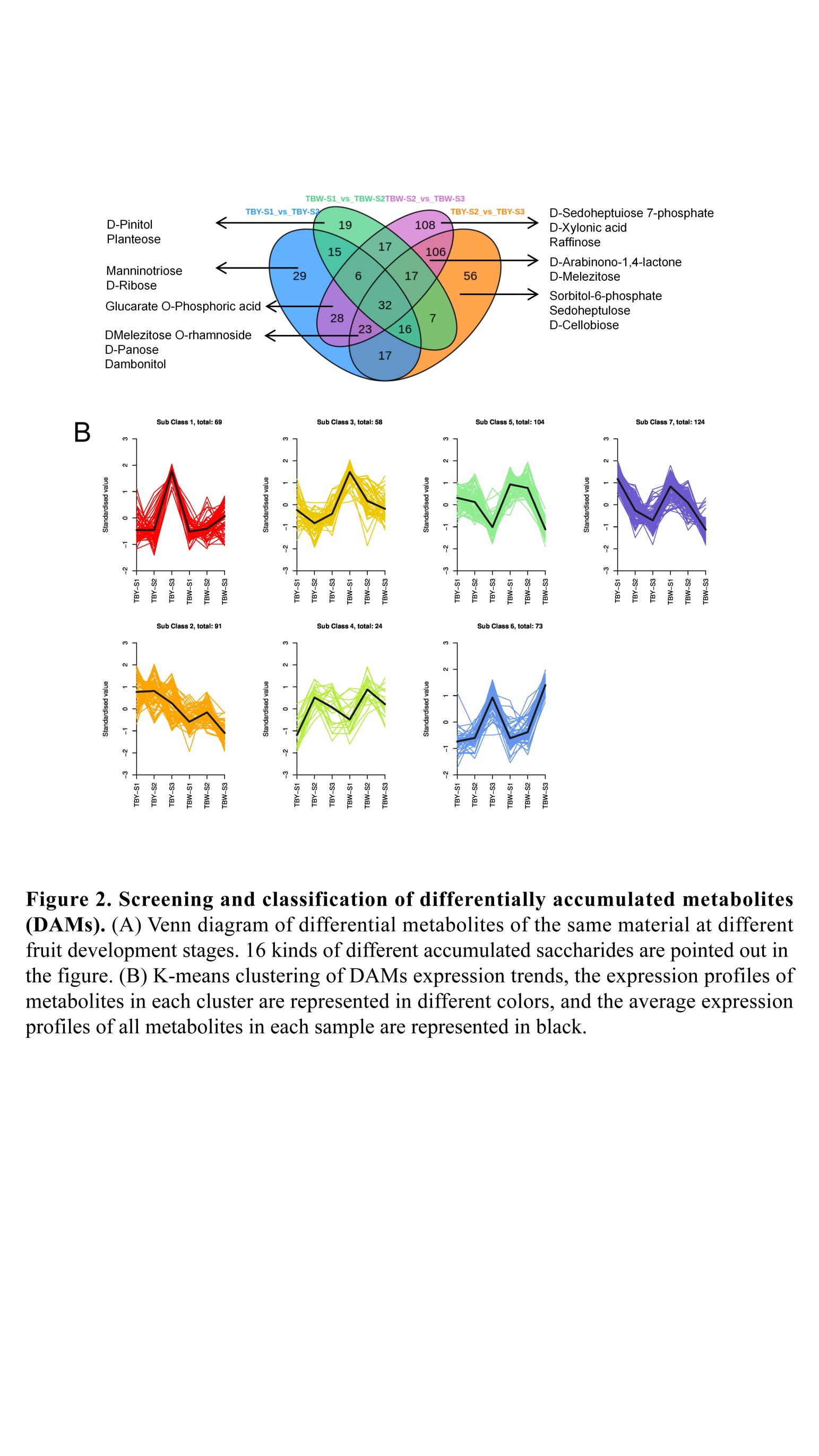


**Figure S1.** Venn diagram of differentially accumulated metabolites (DAMs) of the same material at different developmental stages. Among them, 16 differential accumulation of saccharides were pointed out by the red arrows.


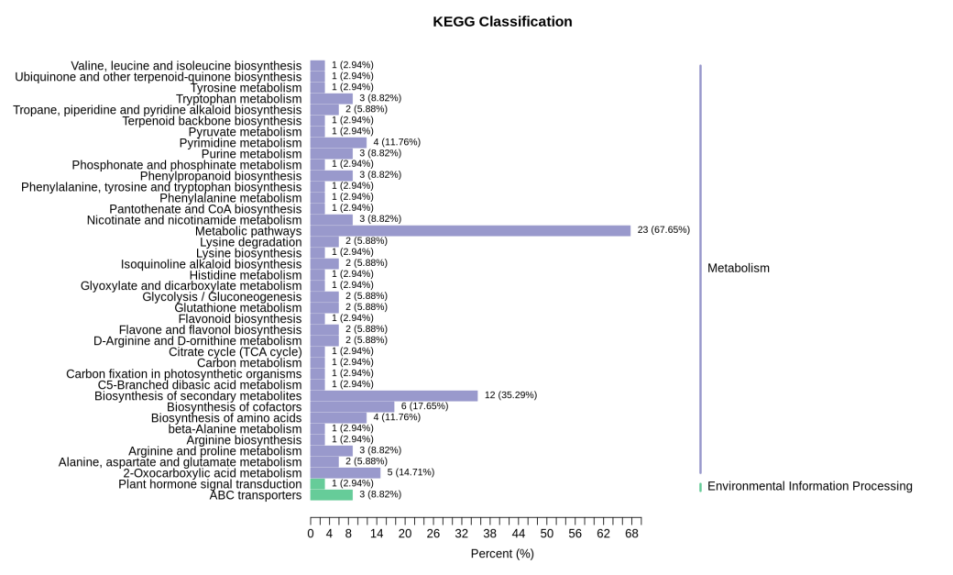


**Figure S2.** KEGG enrichment of DAMs between TBY and TBW at S1 stage.


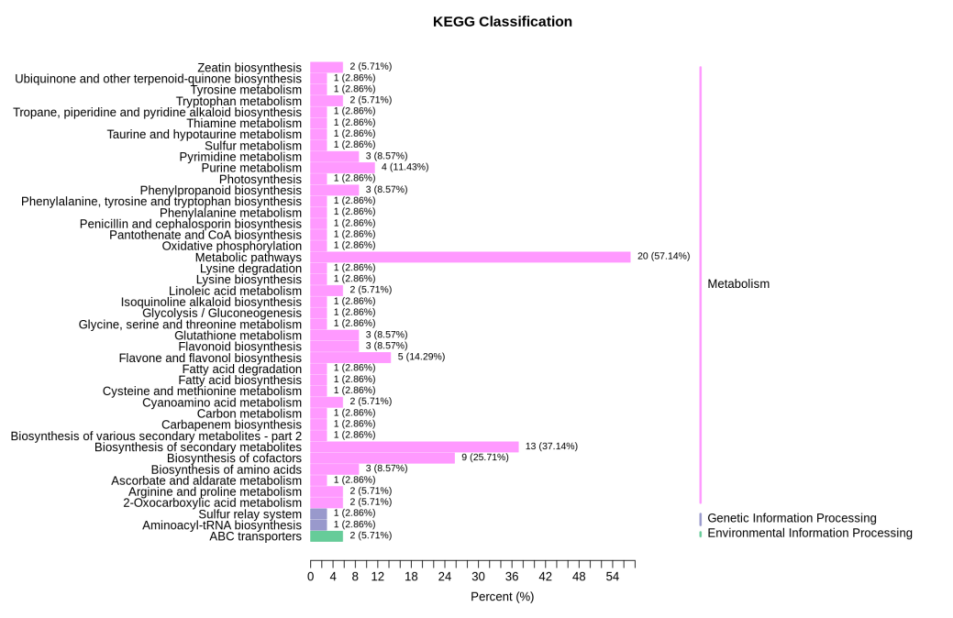


**Figure S3.** KEGG enrichment of DAMs between TBY and TBW at S2 stage.
